# Laparoscopic cholecystectomy in a patient with situs inversus totalis after videolaparoscopic sleeve—Case report

**DOI:** 10.1016/j.ijscr.2020.04.045

**Published:** 2020-05-19

**Authors:** Fernando Ponce Leon, Mariana H. Fiorencio, Camilla P. Leal, André R. Santos

**Affiliations:** a Department of Surgery, Federal University of Rio de Janeiro, Brazil; b Clementino Fraga Filho University Hospital (HUCFF - UFRJ), Brazil

**Keywords:** Situs inversus, Cholecystectomy, Obesity, Cholelithiasis, Videolaparoscopy

## Abstract

**Introduction:**

Situs inversus totalis is the technical term used when there is a complete transposition of all organs to the opposite side referring to classical embryogenic orientation. Laparoscopic cholecystectomy is one of the most performed surgeries in the world.

**Presentation of case:**

We report the case of a 61-year-old female patient who had previously undergone videolaparoscopic gastroplasty 7 months ago, who developed refractary biliary colic and underwent videolaparoscopic cholecystectomy.

**Discussion:**

The surgical technique is challenging in these cases, specially because of the mirror findings in relation to the original technique. An additional challenge in this case was the fact that the patient had a previous laparoscopy due to gastroplasty performed. The presence of adhesions related to the surgery hardly interfered with the Calot triangle dissection, but the way they presented themselves required the attention of the entire surgical team.

**Conclusion:**

Although uncommon, laparoscopic cholecystectomy can be performed in a situs inversus patient with ease, even in the presence of a previous surgical approach.

## Highlights

• Laparoscopic cholecystectomy in a patient with situs inversus totalis after videolaparoscopic sleeve - Case Report.
• A unique case of a surgery at a *situs inversus totalis* patient.
• A new laparoscopic procedure at a patient submitted to a preview lap gastroplasty.
• A complex surgery made in a latin american university hospital.

## 1 Introduction

Situs inversus totalis is the technical term used when there is a complete transposition of all organs to the opposite side referring to classical embryogenic orientation [1]. Its incidence varies widely in the scarce literature on the subject, ranging from 1: 10,000 to 1: 20,000 individuals [1,2]. Laparoscopic cholecystectomy is one of the most performed surgeries in the world, with its technique described in the late 1980s [3]. There is no evidence of increased incidence of cholelithiasis in patients with situs inversus totalis, but anatomical changes of the biliary tract are expected due to differentiation of embryological growth [2,4]. We report the case of a 61-year-old female patient who had previously undergone videolaparoscopic gastroplasty 7 months ago, who developed refractary biliary colic and underwent videolaparoscopic cholecystectomy. Our work has been reported in line with the SCARE criteria [5].

## 2 Case report

**Fig. 1:**
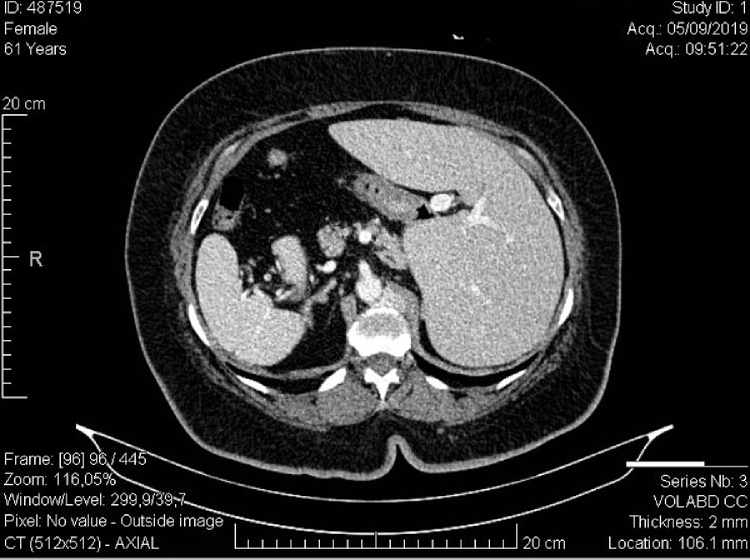
CT scan of the upper abdomen of the patient.

LSB, female, 61 years old, weight 108 kg, height of 1.62 m (BMI 41.2), retired, resident of Rio de Janeiro, obese, hypertensive on enalapril, atenolol, hydralazine and spironolactone, non-insulin dependent diabetic on metformin, dyslipidemic on simvastatin, former smoker (5 packs / year), followed at the obesity outpatient clinic of the Clementino Fraga Filho University Hospital (HUCFF - UFRJ), with indication for videolaparoscopic gastroplasty. She was then submitted to the proposed procedure on 06/11/2019, and vertical gastroplasty was chosen because she had situs inversus totalis previously discovered in non-surgical medical research (Fig. 1). During postoperative follow-up, the patient adhered to the recommendations of the Nutrition and Endocrinology teams, there was a significant reduction in antihypertensive medications and oral antidiabetic drugs. Estimated 6-month weight loss of 20 kg (Weight 88 kg / BMI 33.5).

**Fig. 2:**
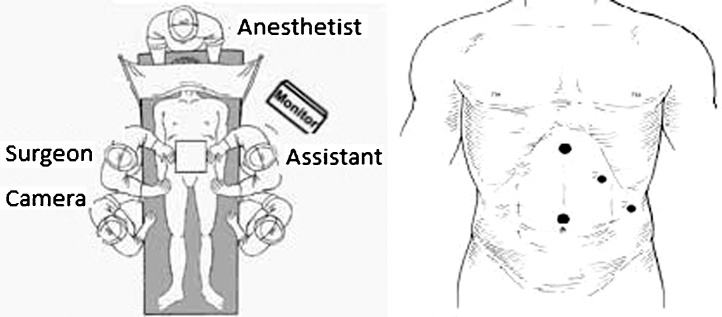
Team positioning and trocar placement.

**Fig. 3:**
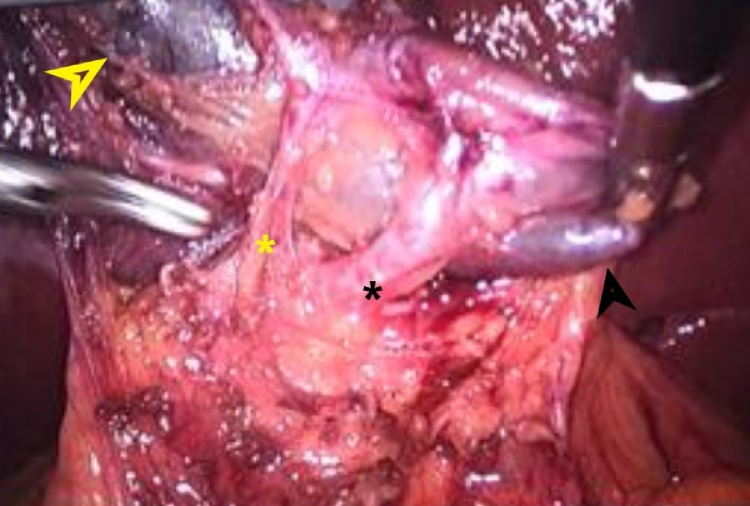
Laparoscopic findings; yellow arrow: body of the gallbladder. Black arrow: Hartmann’s pouch. Yellow asterisk: cistic artery. Black asterisk: Cistic duct.

Two months after surgery, the patient reported colic abdominal pain, with predominance of it in the left upper quadrant, associated with nausea, of weekly frequency, usually related to copious eating. Total abdomen ultrasound performed prior to obesity surgery revealed moderate hepatic steatosis and cholelithiasis (asymptomatic patient at time of examination). After manifestation of symptoms, videolaparoscopic cholecystectomy was indicated. Procedure performed on 12/17/2019. Fig. 2 shows the positioning of the team and equipment. Fig. 3 demonstrates the laparoscopic finding. The patient presented satisfactory evolution in the immediate postoperative period, was discharged in 24 h and has been asymptomatic since then, being followed by the teams of General Surgery, Nutrology and Endocrinology.

## 3 Discussion

The description of situs inversus totalis was described in 1600 by Fabricius et al. [6], with the first described laparoscopic cholecystectomy performed on a patient with this condition in 1991 [3]. Until that moment, the use of laparoscopy was condemned, since there was no guaranteed safety of adequate bile duct evaluation [3]. Since then, with advances in surgical technique, instrumental and learning curve of surgeons, some case reports and systematic reviews of few cases have been performed on this theme [7,8].

In the case of this patient, her anatomical alteration was already known, since she had undergone bariatric surgery seven months ago. Two months after surgery, the symptoms of left hypochondrium pain after fat feeding raised the possibility of symptomatic cholelithiasis. However, in previously healthy patients, this diagnosis can be challenging, since there are few surgical differential diagnoses associated with acute pain in the left hypochondrium [8]. The use of complementary imaging exams such as total abdomen ultrasound and abdominal computed tomography are crucial for diagnostic elucidation [8].

The surgical technique is challenging in these cases. From the placement of the trocars, in mirror in relation to the original technique, until the dissection of the Calot triangle and its detachment from the hepatic bed. The presence of anatomical variations is common in these cases and may reach up to 25% of the time according to Polguj et al. [9]. Therefore, the traction of the Hartmann pouch to the left iliac fossa with the right hand and the meticulous dissection of the Calot triangle with the left hand must be performed in order to be safe in obtaining the critical safety view [10]. and proper clipping of the cystic duct and cystic artery.

An additional challenge in this case was the fact that the patient had a previous laparoscopy due to gastroplasty performed. Wittgrove et al. [11] were the first surgeon to describe a videolaparoscopic gastric bypass in 1998 and since then several authors have been describing several techniques to try to standardize the best technique in these cases [[12], [13], [14]]. The presence of adhesions related to the surgery hardly interfered with the Calot triangle dissection, but the way they presented themselves required the attention of the entire surgical team. The patient's weight loss in the interval between surgeries also helped, since with a lower amount of visceral fat existing, the manipulation, identification and dissection of vascular and biliary structures occurred less difficultly.

## 4 Conclusion

Although uncommon, laparoscopic cholecystectomy can be performed in a situs inversus patient with ease, even in the presence of a previous surgical approach, regarding the correct technique, planning of the surgical act and knowledge of the surgical staff.

## Declaration of Competing Interest

There isn’t any conflict of interest.

## Funding

There weren’t any kind of funding for this paper.

## Ethical approval

This a case report from a patient submitted to an elective surgery. The patient agreed with the publication in its consent form. There is no need for an ethical approval evaluation.

## Consent

Written informed consent was obtained from the patient for publication of this case report and accompanying images. A copy of the written consent is available for review by the Editor-in-Chief of this journal on request.

## Author contribution

Ponce Leon, Fernando – author, writer (original draft), conceptualization, visualization.

Fiorencio, Mariana H – assistant in the surgery, writer (investigation).

Leal, Camilla K P – assistant in the surgery, writer (methodology).

Santos, André R – Main surgeon, writer (project administration).

## Registration of research studies

This is not a research study. It’s a case report only.

## Guarantor

Fernando Ponce Leon.
